# Survey dataset on pathologic internet use, problematic cell phone use and gambling through EUPI-A, CERM and SOGS-RA scales in high technological schools in the north of Spain

**DOI:** 10.1016/j.dib.2019.104121

**Published:** 2019-06-10

**Authors:** Francisco Javier Lena-Acebo, María Elena García-Ruiz

**Affiliations:** Universidad de Cantabria, Spain

**Keywords:** Adolescent, Behaviour, Education, SOGS-RA, EUPI-A, CERM

## Abstract

The incursion of Information Technologies in the field of education is an undeniable fact that today includes immersion in media education in the classroom. The increase in the use of ICTs in the classroom has raised a concern about the possible impact on the prevalence of problems associated with problematic use of the Internet. This article presents data on an exploratory cross-sectional non-experimental design carried out based on a non-probability prepositive sample through the application of an ad-hoc developed questionnaire. The data in this article correspond to the responses of 832 students of Compulsory Secondary Education in three educational centres in the Autonomous Community of Cantabria, Spain. Two of the educational centres are privately owned, and one of them is public. One of the private centres uses educational methodologies based on the high use of information and communication technologies, as each student uses a personal computer in the classroom. The questionnaire used includes EUPI-A, SOGS-RA, and CERM scales. The questionnaire also includes questions about time spent on internet use and sports betting and gambling or game participation. The data are of interest of researchers in developmental, educational, and behavioural psychology and in statistical scale development.

**Table:** Specifications table

Subject area	*Social Sciences*
More specific subject area	*Quantitative Psychology*
Type of data	*Tables, figures and text file*
How data was acquired	*Field Survey using questionnaire*
Data format	*Raw, partial analysed (descriptive statistics)*
Experimental factors	*Convenience sampling of some selected secondary schools in Cantabria, Spain. Non response observations have been removed.*
Experimental features	*Sample selection of the responses of secondary education students from structured questionnaire that includes three scales: EUPI-A (PIUS-A), CERM, SOGS-RA to measure the implication of high technology educational methodology on secondary schools.*
Data source location	*Cantabria, Spain*
Data accessibility	*All the data are included in this data article*

**Table:** 

**Value of the data** • The data provide descriptive statistics of the samples in three types of educational centres, public with traditional educational methodologies, private with traditional educational methodologies, and private with educational methodologies based on the high implementation of information and communication technologies. • The data can provide insight into the similarities and differences in patterns of behaviour on secondary school students when completely analysed. • The data provide new evidence on the functioning of the instruments used -SOGS-Ra, CERM, EUPI-A, in different segmentable samples by age, type of educational centre, gender, or educational level so that researchers can gain more insight on the instruments used for data collection. • The data allow the comparison between the effects of the application of educational methodologies with high involvement in information and communication technologies and traditional methodologies. • The central theme is the study of adolescent behaviour and the behavioural implications on the intensive use of ICTs methodologies so that the data could be very useful in some research areas such as: child behaviour, adolescent behaviour, adolescent and child education, mental health, psychopathology, developmental psychology, and clinical psychology. The questionnaire can be adapted or adopted in order to apply to longitudinal studies.

## 1 Data

In 2009, J.L. Rodríguez Zapatero – President of the Spanish Government – announced the ICT Plan with the aim of updating education by the use of portable devices. This ICT Plan includes the use of a device per student for educational use in a model called one-to-one. The implementation of this ICT plan was uneven, mainly due to the political particularities of regional governments, difficulties in the expansion of internet broadband, the complexity of the management of based laptops in schools and the lack of budget aggravated by the economic crisis that the country suffered. For those reasons, the plan was not fully implemented in schools. This movement of educational renewal collided head-on with the costs of acquiring technology -devices, network infrastructure and software, the low preparation of educational content by publishers, and the economic crisis that meant a sharp budget cut in both public and subsidised schools.

In spite of this, in recent years, the process of methodological change based on the adaptation of New Technologies and the Internet in the classroom has undergone a strong revolution in Spain. This revolution takes place, in part, thanks to the proliferation of devices such as Chromebook or iPad devices, with cheaper costs for the end user, simplicity of use by the student, and simple management by the school. In the case of Chromebook devices, in the last two years, there have been many educational experiences that have been carried out jointly by schools, Google - the company responsible for the platform of administration and service G-Suite for Education and the ChromeOS system, the fundamental basis for the operation of Chromebook, and the respective regional Ministries of Education. These experiences are aimed at introducing Chromebook as a digital platform for content managed and provided through G-Suite and complemented by the use of digital licenses of online course books. In the Autonomous Community of Cantabria, where the implementation of the ICT 2.0 plan was not completed at all, there has been a trend in recent years that advocates methodological change and the introduction of ICT in the classroom. Nowadays (2019), only the reference educational centre of this study presents the use of a Chromebook device in one-to-one mode at all levels of Secondary Education in Cantabria.

Despite the fact that at the time of the study (2016) the implementation of digital contents and Chromebook-managed devices was already taking place in a few schools in other communities of the country, the implementation of the one-to-one model (a device for each student) was a new feature in the community of Cantabria. On this date, only the educational centre of the study had this model of classroom work for all levels of ESO. The choice of this educational centre was justified by the purpose of the study and, therefore, of convenience.

The choice of the other two centres that make up the study is determined by the choice of both public and private centres in the degree of an economic concert by the Ministry of Education. For both cases, the choice of the sample limits the possibilities to those centres that use classroom work methodologies characterised by the use of traditional resources and methodologies: use of physical books, carrying out tasks based on explanation, or group work without focusing their development on the use of the Internet or online tools, except for those subjects and tasks where their use is necessary (subjects such as programming, computing, or the carrying out of certain school tasks).

The data in this article correspond to the responses of 832 students of Compulsory Secondary Education in three educational centres in the Autonomous Community of Cantabria, Spain. Two of the educational centres are privately owned, and one of them is public. One of the private centres uses educational methodologies based on the high use of information and communication technologies, as each student uses a personal computer in the classroom. Descriptive details of the sample are shown in Table 1, Table 2, Table 3, Table 4, Table 5.

**Table 1:** School type of respondents.

Gender	Total	Public (Low Tech)	Private	
			Low Tech	Hi Tech
Female	370	127	119	124
Male	462	153	146	163
Total	832	280	265	287

**Table 2:** Age (Mean) by educational level of respondents.

Level	Total	Public (Low Tech)	Private	
			Low Tech	Hi Tech
ESO1	12.4	12.5	12.2	12.3
ESO2	13.3	13.4	13.2	13.2
ESO3	14.5	14.6	14.5	14.4
ESO4	15.3	15.4	15.2	15.3
Total	13.7	13.8	13.7	13.7

**Table 3:** Gender of respondents.

Gender	Total	Public (Low Tech)	Private	
			Low Tech	Hi Tech
Female	370	127	119	124
Male	462	153	146	163
Total	832	280	265	287

**Table 4:** Age of respondents.

Age	Total	Public (Low Tech)	Private	
			Low Tech	Hi Tech
12	148	37	54	57
13	236	88	73	75
14	215	81	61	73
15	172	51	62	59
16	52	19	13	20
17	8	3	2	3
18	1	1	0	0

**Table 5:** Crosstabulation educational level and gender.

Level	Total		Public (Low Tech)		Private			
					Low Tech		Hi Tech	
	♀	♂	♀	♂	♀	♂	♀	♂
ESO1	88	129	28	42	33	37	27	50
ESO2	115	132	48	52	32	42	35	38
ESO3	114	102	36	32	35	37	43	33
ESO4	53	99	15	27	19	30	19	42
Total	370	462	127	153	119	146	124	163

In order to carry out the study, and in the absence of a consensual or firm bibliographic definition, the operational definition of the *"high use of information and communication technologies at classroom"* has been estimated for this study as.

“the use of ICT technologies in a one-to-one approach in which each student has his or her own device for the course of the classes, the online educational contents, and the necessary software to carry out the academic tasks with the use of these devices in practically the totality of the classroom subjects, reaching an average use superior than 4 h a day on average.”

The centres that show a "low technology use in the classroom" show a use inferior to the 2 h a day on average.

The data were collected by the use of a questionnaire that includes EUPI-A [1], [2], SOGS-RA [3], [4], [5], [6] and CERM scales [7], [8], [9], [10], [11], [12]. The questionnaire also includes questions about age, gender, internet use on classroom and out of classroom, as well as sports betting and gambling prevalence. Reliability of scales are shown on Table 6.

**Table 6:** Scales reliability.

Scale	Total	Public	Private		
		Low Tech	Low Tech	Hi Tech	Theoretical
EUPI-A	0.798	0.783	0.811	0.793	0.820
SOGS-RA	0.766	0.765	0.853	0.616	0.805
CERN	0.760	0.728	0.785	0.761	0.800

## 2 Experimental design, materials, and methods

The main objective of the work was to research the possible explanatory interrelation between the high use of technology in the classroom and potentially problematic behaviours in adolescents through a study not carried out under experimental conditions -without manipulation of dependent variables - in a subset of the population in the age range included in compulsory secondary education. In order to carry out this study, an exploratory cross-sectional non-experimental design was carried out. This study was based on a non-probability purposive sample, since this is a sample in which the centres were classified by type for the purpose of the study and the students were not randomly sampled when all the students participated, focused on the population of Secondary Education students in the Autonomous Community of Cantabria through the application of an ad-hoc developed questionnaire, built specifically for this study.

The study included a sample of 832 students of Compulsory Secondary Education between the ages of 12 and 17 (M = 13.72, SD = 1.21), of which 370 were girls (44.47%) and 462 boys (55.53%), from different public and private (subsidised) educational centres in the Autonomous Community of Cantabria (a population of 22,003 pupils). One of the private centres is immersed in an Educational Innovation Programme (PIIE programme) of the Government of Cantabria and is characterised by the intensive use of technology in the classroom: students use a Chromebook device as a working tool, use digital books and have constant access to the Internet - albeit limited and secure. All the centres considered in the sampling present several lines and do not show segregation of the students.

Educative Centre A, (Private, educational methodologies include High Technology use in the classroom, n = 287). Religiously-owned centre subsidised by the Department of Education of the Government of Cantabria for the teaching of the levels of Compulsory Secondary Education, which also teaches the levels corresponding to the Baccalaureate. All the answers were obtained through the online questionnaire posed by the researchers. The centre, located in an area of the city with a population of average socioeconomic characteristics, represents one of the most advanced bets of the Regional Ministry of Education as one of the participants in the PIIE Programme - Integrated Programme of Educational Innovation - [13], which aims at the evolution of education through methodological reform in the classroom. The commitment to technology in the centre is strong, along with the reform of the programming of subjects, its staff follows an intense training program in new teaching methodologies and use of ICTs in the classroom as a teaching medium and, since its implementation, has provided students with a device (a Chromebook) in possession. The participation of ESO students was very high, participating in all the courses of the four educational levels, reaching a participation of 287 students. In this case, the centre studies chose a Chromebook device, which includes content and tasks distributed through several tools such as Moodle or Google Classroom, digital textbooks and software (office suites and mail systems, such as Google Drive or Gmail in the case of G-Suite).

For the choice of the other two educational centres, the characteristics of the main centre were taken into consideration due to the similarity, focusing on the existence of three or more courses per level. Thus, in the case of subsidised or private centres, it was limited to 9 of the 42 possible samples to be considered. In the case of the public centre, this limited the possibilities to 40 of the 47 possible centres. The choice of the representative centre of each category was made randomly.

Educative Centre B, (Private, classic educational methodologies with Low Technology use in the classroom, n = 265). A religiously-owned centre subsidised by the Department of Education of the Government of Cantabria for the teaching of the levels of Compulsory Secondary Education. The centre is located in an area of the city with a medium-high purchasing power, has the technological means in accordance with the commitment made by the Ministry of Education of the Government of Cantabria for the use of ICT in the classroom, without being integrated as a PIIE centre. This commitment includes the use of pedagogical means, such as digital blackboards, but not the integration of a specific device for students or the use of specific digital didactic means. The participation of ESO students was high, reaching a participation of 265 students. All student responses were collected through the printed questionnaire and were manually transcribed for analysis.

Educative Centre C, (Public, classic educational methodologies with Low Technology use in the classroom, n = 280). A Public Centre belonging to the Department of Education of the Government of Cantabria located in an urban municipality near the capital of the Autonomous Community. This centre offers ESO studies and develops different educational plans, including the ICT plan, which provides specific computer rooms and computers with a projector in the classrooms. The use of new technologies and Internet at the centre is, therefore, quite limited, counting the aforementioned rooms with 16 and 18 connected computers for the more than 500 students that represent the total number of enrolled. The participation of the students of this centre covers the stages of ESO, with a participation close to 75% of the Secondary Education students. All the answers were obtained through the paper questionnaire and were transcribed individually for analysis. The questionnaire was a designed ad-hoc and included EUPI-A, SOGS-RA, and CERM scales.

**Fig. 1:**
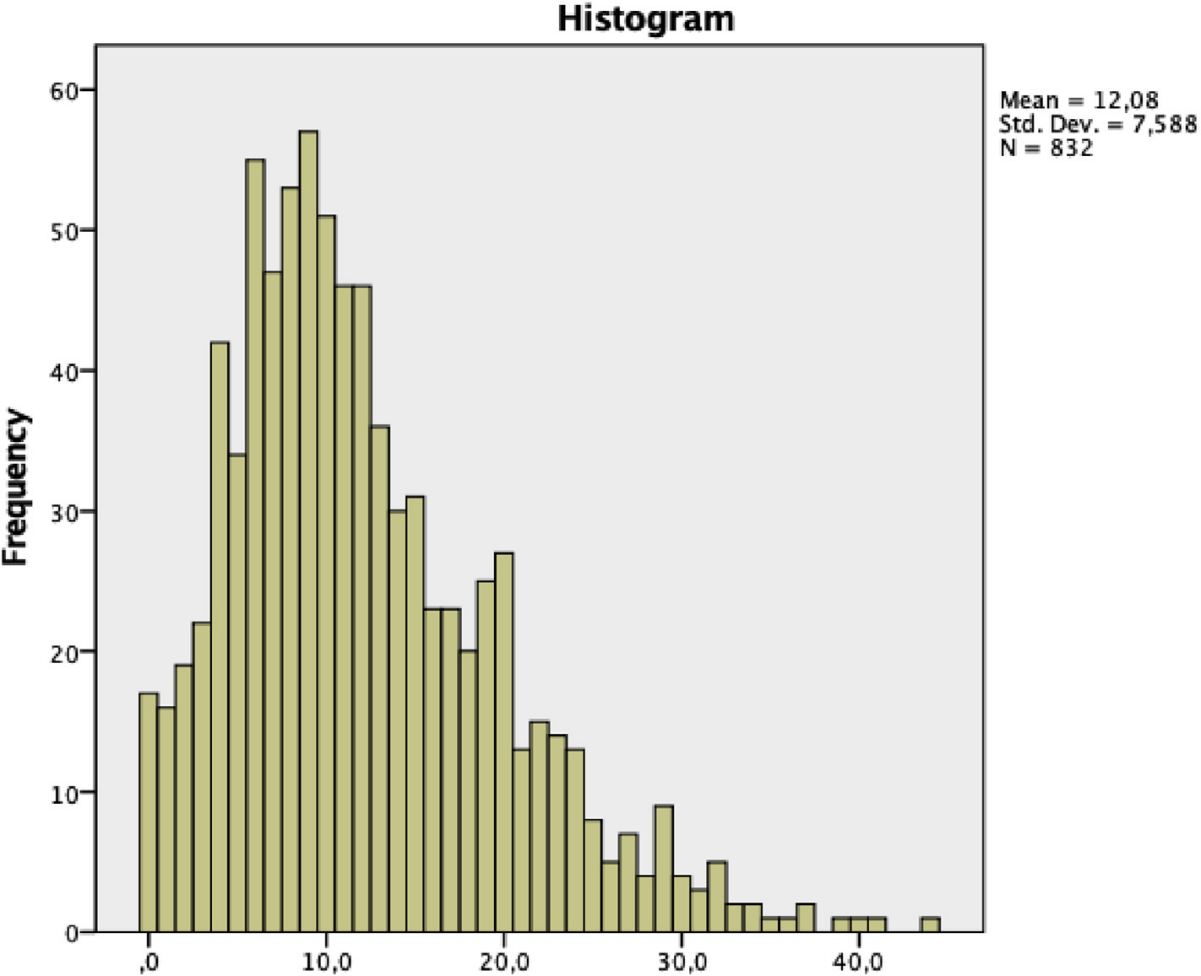
EUPI-A scores histogram.

EUPI-A scale. Developed with the aim of becoming a screening scale of problematic Internet use among adolescents, developed according to the diagnostic criteria collected in the DSM-V for gambling and gambling disorder based on the Internet, as well as the instruments and studies previous to the date of its construction. It includes 11 first-person statements, relating to a single dimension, with a Likert type response format of 5 options in a range of values from 0 -Nothing agree-to 5 -Totally agree-. The scale allows discrimination in its application between Moderate Internet Use and Problematic Internet Use. Originally, in its validation, the questionnaire yielded a Cronbach alpha reliability (0.820), establishing its cut-off point at 16 points [1]. Descriptive details of the sample on EUPI-A scale are shown in Fig. 1 and Table 7, Table 8, Table 9, Table 10.

**Table 7:** EUPI-A Scores summary statistics.

Level	Total	Public (Low Tech)	Private	
			Low Tech	Hi Tech
Mean	12.084	10.754	12.483	13.014
Median	11	10	11	11
Standard Deviation	7.5878	7.1652	7.9981	7.4435
Variance	57.547	51.340	63.970	55.405
Skewness	0.936	1.183	0.827	0.860
Kurtosis	0.887	1.984	0.568	0.555
Range	44	40	41	44
Minimun	0	0	0	0
Maximum	44	40	41	44
Percentile 25	10,054.0	6	6	8
50	7	10	10	11
75	11	14	18	18

**Table 8:** EUPI-A Scores summary statistics by educational level.

Level	Total			
	ESO1	ESO2	ESO3	ESO4
Mean	10	12.178	12.468	14.362
Median	9	11	11	13.5
Standard Deviation	6.7474	7.9705	8.0301	6.6875
Variance	45.528	63.529	64.483	44.723
Skewness	1.227	0.913	1.053	0.548
Kurtosis	1.9	0.555	1.361	0.181
Range	35	40	44	36
Minimun	0	0	0	1
Maximum	35	40	44	37
Percent.25	6	6	7	9
50	9	11	11	13.5
75	13	16	17	19

**Table 9:** Summary statistics of the EUPI-A scores by educational centre and educational level.

Level	Public (Low Tech)				Private							
					Low Tech				Hi Tech			
	ESO1	ESO2	ESO3	ESO4	ESO1	ESO2	ESO3	ESO4	ESO1	ESO2	ESO3	ESO4
Mean	10.629	11.170	9.279	12.357	9.686	11.770	14.333	14.837	9.714	13.973	13.553	15.361
Median	9	10	7.5	12.5	8	10	13	14	8	12	12	14
Standard Deviation	7.1529	7.7382	7.0602	5.5252	7.2202	8.2438	8.1396	7.2496	5.9224	7.8084	7.9907	6.7553
Variance	51.164	59.880	49.846	30.528	52.132	67.960	66.254	52.556	35.075	60.971	63.851	45.634
Skewness	1.349	1.298	1.385	0.020	0.908	0.924	0.954	0.742	1.539	0.534	1.090	0.408
Kurtosis	2.007	2.325	2.786	−0.735	0.631	0.493	1.004	0.676	3.983	−0.746	1.699	−0.487
Range	34	40	34	23	30	39	39	34	35	32	42	30
Minimun	0	0	0	1	0	0	2	3	0	0	2	3
Maximum	34	40	34	24	30	39	41	37	35	32	42	33
Percentile 25	6	6	4	9	4	6	8	10	6	8	8	10
50	9	10	7.5	12.5	8	10	13	14	8	12	12	14
75	13	14	12	17	14	16	19.5	19	12	20	19	20

**Table 10:** Mean, median and standard deviation of all EUPI-A scale questions obtained from the respondents.

Question	Total			Public (Low Tech)			Private					
							Low Tech			Hi Tech		
	Mean	Median	SD	Mean	Median	SD	Mean	Median	SD	Mean	Median	SD
1	2.476	2	1.301	2.429	2	1.366	2.521	3	1.326	2.481	2	1.211
2	1.135	1	1.267	0.954	1	1.230	0.977	1	1.168	1.456	1	1.332
3	1.397	1	1.366	1.196	1	1.262	1.411	1	1.454	1.578	1	1.359
4	1.419	1	1.234	1.179	1	1.122	1.392	1	1.260	1.679	1	1.266
5	1.125	1	1.268	0.921	0	1.198	1.245	1	1.310	1.213	1	1.274
6	0.918	0	1.288	0.743	0	1.188	1.019	0	1.366	0.997	0	1.294
7	0.286	0	0.736	0.268	0	0.750	0.321	0	0.783	0.272	0	0.676
8	0.553	0	1.042	0.429	0	0.921	0.634	0	1.131	0.599	0	1.059
9	0.924	1	1.089	0.843	1	1.086	0.981	1	1.106	0.951	1	1.076
10	0.953	1	1.206	0.807	0	1.163	1.004	1	1.275	1.049	1	1.173
11	0.898	0	1.266	0.986	0	1.325	0.977	0	1.294	0.739	0	1.167

**Fig. 2:**
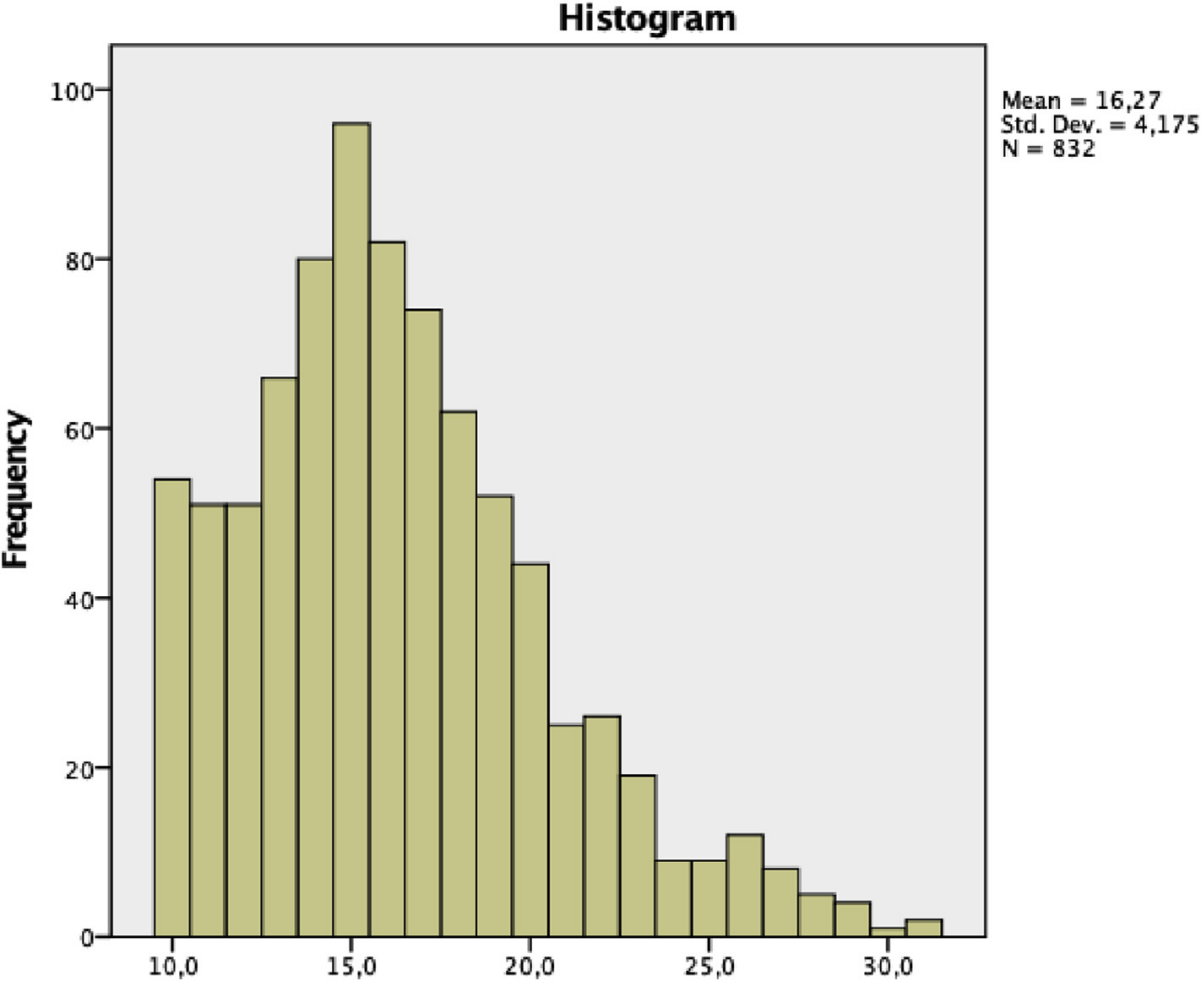
CERM scores histogram.

CERM Scale, Mobile-Related Experience Questionnaire. CERM is, together with CERI -Internet-Related Experience Questionnaire-, an adaptation of the PRI [8], [9] questionnaire that comprises 10 Likert type items with four possible answers in increasing order according to intensity and that contemplates two factors: the existence of conflicts and the communicational (sic) and emotional use. This questionnaire shows good overall reliability, reaching a Cronbach alpha of .805 in its development and allows to estimate, continuously, the degree of problems present in the participants. Occasionally, the use of non-hierarchical cluster analysis can be used to obtain cut-off scores in order to determine the existence of homogeneous groups of responses. Descriptive details of the sample on the CERM scale are shown in Fig. 2 and Table 11, Table 12, Table 13, Table 14.

**Table 11:** CERM Scores summary statistics.

Level	Total	Public (Low Tech)	Private	
			Low Tech	Hi Tech
Mean	16.269	15.714	16.687	16.425
Median	16	15	16	16
Standard Deviation	4.1751	3.9026	4.4000	4.1754
Variance	17.432	15.230	19.360	17.434
Skewness	0.750	0.656	0.799	0.725
Kurtosis	0.454	0.148	0.481	0.470
Range	21	18	21	21
Minimun	10	10	10	10
Maximum	31	28	31	31
Percentile 25	13	13	14	13
50	16	15	16	16
75	19	19	119	19

**Table 12:** CERM Scores summary statistics by educational level.

Level	Total			
	ESO1	ESO2	ESO3	ESO4
Mean	14.959	16.619	16.269	17.572
Median	15	16	16	17
Standard Deviation	4.0726	4.3131	4.1002	3.6857
Variance	16.586	18.603	16.811	13.584
Skewness	1.096	0.602	1.01	0.562
Kurtosis	1.102	0.059	1.451	0.26
Range	19	20	21	19
Minimun	10	10	10	10
Maximum	29	30	31	29
Percent.25	12	13	14	15
50	15	16	16	17
75	17	19	18	20

**Table 13:** Summary statistics of the CERM scores by educational centre and educational level.

Level	Public (Low Tech)				Private							
					Low Tech				Hi Tech			
	ESO1	ESO2	ESO3	ESO4	ESO1	ESO2	ESO3	ESO4	ESO1	ESO2	ESO3	ESO4
Mean	15.057	16.25	14.926	16.81	15.2	16.581	17.417	17.898	14.649	17.164	16.382	17.836
Median	14	16	14	17	15	16	17	17	15	17	16	17
Standard Deviation	4.2559	4.2791	3.3293	2.6709	4.3891	4.4136	4.1034	4.3265	3.612	4.259	4.409	3.7112
Variance	18.113	18.311	11.084	7.134	19.264	19.48	16.838	18.719	13.046	18.139	19.439	13.773
Skewness	1.049	0.602	0.414	0.174	1.113	0.746	1.209	0.486	1.063	0.496	1.039	0.474
Kurtosis	0.725	−0.126	−0.621	−0.247	1.19	0.522	1.447	−0.19	1.325	0.047	1.456	0.098
Range	18	17	13	12	19	20	20	18	16	19	21	18
Minimun	10	10	10	11	10	10	11	10	10	10	10	11
Maximum	28	27	23	23	29	30	31	28	26	29	31	29
Percent.25	12	13	12	15	12	14	15	15	12	14	14	15
50	14	16	14	17	15	16	17	17	15	17	16	17
75	17	19	17.5	19	18	19	19	20	16	20	18.5	20

**Table 14:** Mean, median and standard deviation of all CERM scale questions obtained from the respondents.

Question	Total			Public (Low Tech)			Private					
							Low Tech			Hi Tech		
	Mean	Median	SD	Mean	Median	SD	Mean	Median	SD	Mean	Median	SD
1	1.182	1	0.4468	1.136	1	0.3634	1.19	1	0.4644	1.22	1	0.4988
2	1.888	2	0.8233	1.746	2	0.769	1.917	2	0.8503	2	2	0.8321
3	1.406	1	0.7014	1.296	1	0.55	1.466	1	0.7595	1.456	1	0.7646
4	1.407	1	0.6858	1.346	1	0.6757	1.411	1	0.6913	1.463	1	0.688
5	1.101	1	0.392	1.111	1	0.4215	1.121	1	0.4278	1.073	1	0.321
6	1.411	1	0.6673	1.336	1	0.6234	1.523	1	0.7255	1.383	1	0.6419
7	2.905	3	1.0117	2.757	3	1.0532	3.049	3	0.9757	2.916	3	0.9859
8	1.624	1	0.8016	1.657	1	0.8319	1.665	1	0.8068	1.554	1	0.7639
9	1.666	1	0.8434	1.693	1	0.8584	1.683	1	0.838	1.624	1	0.8349
10	1.704	1.5	0.8529	1.636	1	0.8183	1.741	2	0.8876	1.735	2	0.8525

The SOGS-RA questionnaire in its Spanish version [5] is a 12-item dichotomous (Yes/No) questionnaire to assess the presence of problem gambling and risk gambling, derived from the SOGS questionnaire [6] and adapted to the adolescent population. It is capable of achieving reliability, evidenced by the Cronbach alpha coefficient of 0.800. This scale allows the classification of the participants in non-player or without gambling problems, risk player or problematic player according to the cut points, usually those proposed by and used in different studies; 0–1 without gambling problems, 2–3 player at risk, 4 or more problem players. Descriptive details of the sample on the SOGS-RA scale are shown in Table 15, Table 16, Table 17.

**Table 15:** SOGS-RA Scores summary statistics.

Level	Total	Public (Low Tech)	Private	
			Low Tech	Hi Tech
Mean	0.204	0.214	0.192	0.206
Median	0	0	0	0
Standard Deviation	0.8160	0.8363	0.9317	0.6713
Variance	0.666	0.699	0.868	0.451
Skewness	7.003	6.244	7.959	5.320
Kurtosis	63.454	45.703	77.572	34.884
Range	11	8	11	6
Minimun	0	0	0	0
Maximum	11	8	11	6

**Table 16:** OGS-RA Scores summary statistics by educational level.

Level	Total			
	ESO1	ESO2	ESO3	ESO4
Mean	0.166	0.134	0.236	0.329
Median	0	0	0	0
Standard Deviation	0.4084	0.4624	1.135	1.1025
Variance	0.167	0.214	1.288	1.216
Skewness	2.406	4.699	6.441	4.604
Kurtosis	5.275	27.834	47.435	23.565
Range	2	4	11	8
Minimun	0	0	0	0
Maximum	2	4	11	8
Percent.25	12	13	14	15
50	15	16	16	17
75	17	19	18	20

**Table 17:** Data of SOGS-RA scale questions obtained from the respondents.

Question	Total		Public (Low Tech)		Private			
					Low Tech		Hi Tech	
	YES	NO	YES	NO	YES	NO	YES	NO
1	18	814	7	273	5	260	6	281
2	11	821	4	276	7	258	0	287
3	7	825	3	277	4	261	0	287
4	51	781	16	264	10	254	25	262
5	21	811	8	272	6	259	7	280
6	18	814	8	272	2	263	8	279
7	13	819	4	276	4	261	5	282
8	5	827	3	277	2	263	0	287
9	11	821	3	277	4	261	4	283
10	3	829	0	280	1	264	2	285
11	5	827	2	278	2	263	1	286
12	7	825	2	278	4	261	1	286

The questionnaire also included some questions about time spent on internet use and sports betting and gambling or game participation. Data obtained from all respondents are shown in Table 18, Table 19, Table 20.

**Table 18:** Internet use in the classroom.

		<1h	1–2h	2–3h	3–4h	4–5h	>5h
ESO1	Public (LT)	57	10	3	0	0	0
	Private (LT)	47	3	4	7	6	3
	Private (HT)	0	3	5	21	36	12
ESO2	Public (LT)	77	15	3	2	2	1
	Private (LT)	38	33	1	1	1	0
	Private (HT)	0	1	5	10	26	31
ESO3	Public (LT)	37	19	6	3	2	1
	Private (LT)	48	11	5	5	2	1
	Private (HT)	1	6	8	6	21	34
ESO4	Public (LT)	27	15	0	0	0	0
	Private (LT)	30	6	1	0	2	10
	Private (HT)	35	19	4	2	1	0

**Table 19:** Internet use outside the classroom.

		<1h	1–2h	2–3h	3–4h	4–5h	>5h
ESO1	Public (LT)	18	27	18	4	2	1
	Private (LT)	24	32	3	6	3	2
	Private (HT)	11	23	25	10	5	3
ESO2	Public (LT)	16	36	20	12	5	11
	Private (LT)	11	27	21	7	5	3
	Private (HT)	1	11	22	27	5	7
ESO3	Public (LT)	9	23	25	10	5	4
	Private (LT)	8	20	10	20	8	6
	Private (HT)	6	11	18	20	5	16
ESO4	Public (LT)	1	7	17	9	2	6
	Private (LT)	2	9	9	12	6	11
	Private (HT)	4	8	19	14	8	8

**Table 20:** Respondents who claim to have participated in gambling/sport bets.

		Gambling		Sport Bets	
		♀	♂	♀	♂
ESO1	Public (LT)	0	0	0	1
	Private (LT)	0	2	0	3
	Private (HT)	0	0	0	0
ESO2	Public (LT)	0	1	0	2
	Private (LT)	0	1	1	2
	Private (HT)	0	1	0	2
ESO3	Public (LT)	0	2	1	3
	Private (LT)	1	3	0	5
	Private (HT)	1	5	0	7
ESO4	Public (LT)	0	5	0	5
	Private (LT)	1	3	0	4
	Private (HT)	1	5	1	7
