# Supplementary material for: Survey dataset on pathologic internet use, problematic cell phone use and gambling through EUPI-A, CERM and SOGS-RA scales in high technological schools in the north of Spain
Source: Data Brief. 2019 Jun 10;25:104121. doi: 10.1016/j.dib.2019.104121 (PMC6610689; doi:10.1016/j.dib.2019.104121)
Supplement: Multimedia component 2 [file mmc2.pdf]

Please indicate in each case the correct answer:: ☒.
Remember, this questionnaire is **anonymous**.
Don't write your name

1.Age (Years) ▭ **2. Gender:** Male ☐ Female ☐

3ª. ¿Course?: ☐ 1 ESO ☐ 3 ESO ☐ 2 ESO ☐ 4 ESO

4. How many hours, on average, are you connected to the Internet in class per day? (approximately)
Indicate how many hours you spend connected to the Internet in the classroom each day (approximately)

☐ Less than 1 hours ☐ Between 3 y 4 hours
☐ Between 1 y 2 hours ☐ Between 4 y 5 hours
☐ Between 2 y 3 hours ☐ Between de 5 hours

5. How many hours, on average, are you connected to the internet outside of school per day? (approximately)

Indicate how many hours you spend connected to the Internet when you are not in class each day (at home, from your mobile,...)

☐ Less than 1 hours ☐ Between 3 y 4 hours
☐ Between 1 y 2 hours ☐ Between 4 y 5 hours
☐ Between 2 y 3 hours ☐ Between de 5 hours

6. Indicate your degree of agreement with the following statements:

		Not agree at all [1]	[2]	[3]	[4]	Totally Agree [5]
a.	When I connect I feel that time flies and hours pass without realizing	☐	☐	☐	☐	☐
b.	Sometimes I have tried to control or reduce my use of the internet, but I was unable to do so.	☐	☐	☐	☐	☐
c.	In some occasions I have been able to neglect some tasks or perform less (in exams, sports, etc...) by connecting to the internet.	☐	☐	☐	☐	☐
d.	I like to spend more and more hours connected to the internet	☐	☐	☐	☐	☐
e.	Sometimes I get irritated or in a bad mood because I can't connect to the internet or have to disconnect.	☐	☐	☐	☐	☐
f.	I prefer that my parents don't know how much time I spend online because they think it would be excessive.	☐	☐	☐	☐	☐
g.	I have stopped going to sites or doing things that used to interest me in order to be able to connect to the internet.	☐	☐	☐	☐	☐
h.	On occasion I have gotten into trouble or problems because of the Internet	☐	☐	☐	☐	☐
i.	It annoys me to spend hours without connecting to the Internet	☐	☐	☐	☐	☐
j.	When I can't connect I can't stop thinking if I'll be missing something important.	☐	☐	☐	☐	☐
k.	I say or do things on the internet that I would not be able to do/say in person	☐	☐	☐	☐	☐

7. Have you ever bet money on a game of chance (poker, cards, roulette) through the internet YES ☐ NO ☐

8. Have you ever bet money on a game based on sports scores over the internet? YES ☐ NO ☐

9. Read the following statements carefully and answer YES or NO to the questions:

		YES	NO
a.	In the last year, if you've bet money, do you always claim to have won even though you've actually lost it?	☐	☐
b.	In the past year, have you played another day to make up for lost money?	☐	☐
c.	Did gambling in the past year ever cause you problems such as arguments with family and friends, or problems at school or work?	☐	☐
d.	In the last year, have you ever played more than you had planned?	☐	☐
e.	In the last year, have you been criticized for your game or has someone told you that you had a game problem even though you don't think it's true?	☐	☐
f.	In the last year, have you ever felt that you would like to stop playing but don't feel capable of it?	☐	☐
g.	In the past year, have you ever concealed from your family any guards, money, or objects obtained from the game?	☐	☐
h.	In the last year, have you borrowed money to play and not paid it back?	☐	☐
i.	In the past year, have you had any discussions with your family or friends about gambling money?	☐	☐
j.	In the last year, have you ever missed school because of gambling?	☐	☐
k.	In the past year, have you borrowed money or stolen something to play or to pay off debts caused by the game?	☐	☐
l.	In the last year, have you felt bad about the amount bet or what happens when you bet money?	☐	☐

10. Carefully read the following statements regarding mobile phone use and point out the frequency that most closely approximates your reality:

		Never/ Almost never [1]	Sometimes [2]	Quite a few times [3]	Almost always [4]
a.	Have you had the risk of losing an important relationship, a job or an academic opportunity due to the use of mobile phones?	☐	☐	☐	☐
b.	Do you think your academic or work performance has been negatively affected by mobile phone use?	☐	☐	☐	☐
c.	Do you suffer from sleep disturbances due to aspects related to the mobile phone?	☐	☐	☐	☐
d.	Do you feel the need to invest more and more time in your mobile in order to feel satisfied?	☐	☐	☐	☐
e.	Do you stop going out with your friends because you spend more time using your mobile?	☐	☐	☐	☐
f.	To what extent do you feel restless when you do not receive messages or calls?	☐	☐	☐	☐
g.	When you get bored, do you use your mobile as a form of distraction?	☐	☐	☐	☐
h.	How often do you say things on your mobile that you wouldn't say in person?	☐	☐	☐	☐
i.	Do you think life without a cell phone is boring, empty and sad?	☐	☐	☐	☐
j.	Do you get angry or irritated when someone bothers you while using your mobile?	☐	☐	☐	☐

Please check that you **didn't leave any questions unanswered**.

Thank you very much for your participation.
